# ERP Characterization of Sustained Attention Effects in Visual Lexical Categorization

**DOI:** 10.1371/journal.pone.0009892

**Published:** 2010-03-25

**Authors:** Clara D. Martin, Guillaume Thierry, Jean-François Démonet

**Affiliations:** 1 Departament de Tecnologias, Universitat Pompeu Fabra, Barcelona, Spain; 2 INSERM U825, Hôpital de Purpan, Toulouse, France; 3 School of Psychology, Bangor University, Bangor, Wales, United Kingdom; 4 Economic and Social Research Council (ESRC) Centre for Research on Bilingualism in Theory and Practice, Bangor University, Bangor, Wales, United Kingdom; University of Barcelona, Spain

## Abstract

As our understanding of the basic processes underlying reading is growing, the key role played by attention in this process becomes evident. Two research topics are of particular interest in this domain: (1) it is still undetermined whether sustained attention affects lexical decision tasks; (2) the influence of attention on early visual processing (i.e., before orthographic or lexico-semantic processing stages) remains largely under-specified. Here we investigated early perceptual modulations by sustained attention using an ERP paradigm adapted from Thierry et al. [1]. Participants had to decide whether visual stimuli presented in pairs pertained to a pre-specified category (lexical categorization focus on word or pseudoword pairs). Depending on the lexical category of the first item of a pair, participants either needed to fully process the second item (hold condition) or could release their attention and make a decision without full processing of the second item (release condition). The P1 peak was unaffected by sustained attention. The N1 was delayed and reduced after the second item of a pair when participants released their attention. Release of sustained attention also reduced a P3 wave elicited by the first item of a pair and abolished the P3 wave elicited by the second. Our results are consistent with differential effects of sustained attention on early processing stages and working memory. Sustained attention modulated early processing stages during a lexical decision task without inhibiting the process of stimulus integration. On the contrary, working memory involvement/updating was highly dependent upon the allocation of sustained attention. Moreover, the influence of sustained attention on both early and late cognitive processes was independent of lexical categorization focus.

## Introduction

The idea that word processing is autonomous to some extent is an implicit assumption of visual word recognition models [2], [3]. However, the dependency of linguistic processes on attention is still a matter of debate [4]. Attention-dependent effects in visual word recognition have been studied using the classical “overlapping task paradigm” in which two stimuli requiring separate responses are presented rapidly in a row [5]. Even though lexical access is supposedly independent from central attention (cf. classical Stroop effect; [6]), it appears that it is not entirely autonomous and dependent on task overlap [4], lexical skills [7] and attention [8]. The current literature on the role of central attention on lexical processing is therefore partly inconsistent (see [9]). In contrast with many of the previous studies that have used the “overlapping task paradigm”, here we investigated the interplay between attention and lexical processing using an original paradigm, the Hold/Release paradigm [1], [10] in which the engagement of attention is manipulated directly without intervention of any other task.

According to Coull [11], attention can be divided into four sub-processes, which are attentional orientation, selective attention, divided attention and sustained attention. Sustained attention, which will be the focus of the present experiment, refers to the ability to maintain attention to a particular stimulus or location for prolonged periods of time. Reanalysing PET data from nine studies of human visual information processing, Shulman et al. [12] observed that passive viewing and active discriminations of the same stimuli induce significant modulations in early visual cortex. Dealing with the time course of attentional effects, it has been shown that sustained attention can influence processing tasks as early as 60 ms after the onset [13]. As for early ERP components, several of the late ERP responses (such as P3 event and Late Positive Component (LPC)) have been shown to reflect sustained attention [14], [15]. In their review on emotion and attention, Schupp et al. [16] proposed that sustained attentive processing is reflected by the sustained positive slow waves observed in ERP data in the [300–800] ms time window. To our knowledge, the role of sustained attention on lexical processing has not yet been investigated specifically.

Even though the role of attention on word processing has been studied using different imaging techniques, two important questions remain unanswered: (1) While attention can be divided into four sub-processes (orientation, selective attention, divided attention and sustained attention; [11]), it is not clear how the neural mechanisms involved affect word processing. In the present study, we investigated sustained attention effects in a lexical decision task. The importance of sustained attention has been highlighted in several cognitive processes (cf. [13], [16]) but not specifically in lexical processing. We used a lexical decision task within the context of the Hold/Release paradigm [1], [10], [17] so as to investigate the impact of engaging and disengaging sustained attention in a lexical decision task. In addition, we manipulated lexical categorization focus (either Words or Pseudowords) to evaluate if sustained attention interacts with lexical categorization focus or if these two cognitive processes are independently implemented in language processing. In other words, we tested if it is possible to tease apart, in the same experimental paradigm, sustained attention (stimulus actively processed *versus* passively perceived, e.g., [12]) and lexical categorization focus (focusing on different lexical categories of letter sequences, i.e., searching for words or pseudowords in a stream of letter sequences) and if these two types of cognitive processes interact or take place in the brain independently.

(2) The time window in which sustained attention influences word processing remains controversial. Attentional influences on late stages of stimulus processing are compelling as one can voluntarily and consciously ignore a stimulus after it has been identified [18]. However, effects of attention on earlier stages of perception are less well understood [19], [20], [21] and it is debated whether sustained attention can suppress sensory processing altogether [18]. The present study addressed this issue using ERP measures.

In the present study, we capitalized on the exquisite temporal resolution of ERPs to study the sustained attention effects on lexical processing and the interplay of sustained attention and lexical categorization focus [22], [23], using a paradigm developed originally for the auditory modality [1], [10]. The latter studies investigated auditory phonological and lexical-semantic decision in a context where both sustained attention (processing only the first item of a pair and releasing attention or processing the two items of a pair) and levels of linguistic processing (focusing on phonological, grammatical or semantic properties of stimuli) were manipulated. In the present study, we adapted this paradigm to investigate the interplay of sustained attention and lexical categorization of written stimuli. Participants were asked to decide whether visual stimuli presented in pairs pertained to a pre-specified category (e.g., ‘identify a pair of two real words’, i.e., categorization of letter sequences as words). When the first item was incongruent with the target category (e.g., a pseudoword), participants did not need to fully process the second stimulus and could make a decision and respond immediately because the stimulus pair as a whole could never be a target (release condition). On the other hand, when the first stimulus in a pair was congruent with the target category (e.g., a word), participants needed to sustain their attention in order to successfully process the second stimulus before making a decision (hold condition). Thus, both the items in a pair could be under different lexical categorization focus (word or pseudoword) and either under sustained attention or not (See Table 1).

**Table 1:** Experimental conditions.

Task	Item 1	Item 2	Response button	*Lexical categorization focus*	*Sustained attention* condition	Example
“press A for each pair of words, B in any other case”	wwpp	wpwp	ABBB	WWWW	HoldHoldReleaseRelease	ACTEUR – LIONNELISTE – DAEOURNEAINE – REVEILIUSTE – TIATUE
“press A for each pair of pseudowords, B in any other case”	wwpp	wpwp	BBBA	PPPP	ReleaseReleaseHoldHold	NEVEU – RACINECANAL – RERNESFURE – FABLEMEADI – SGINAL

Response sides and tasks were counterbalanced across blocks and participants (w = word and p = pseudoword). Translations: *Acteur* – ‘Actor’; *Lionne* – ‘lioness’, *liste* – ‘list’, *reveil* – ‘alarmclock’, *neveu* – ‘nephew’, *racine* – ‘root’, *canal* – ‘channel’, *fable* – ‘legend’.

This new paradigm allowed us to investigate directly for the first time the ERP correlates of engagement *versus* disengagement of sustained attention during a lexical decision task. The main goal was to investigate the interplay of sustained attention and lexical categorisation focus effects during early and late processing stages of lexical decision. More specifically, we targeted the two following questions: (a) Are early processing stages of lexical decision influenced by sustained attention? (b) If it exists, is this influence modulated by lexical categorisation focus?

## Results

**Figure 1:**
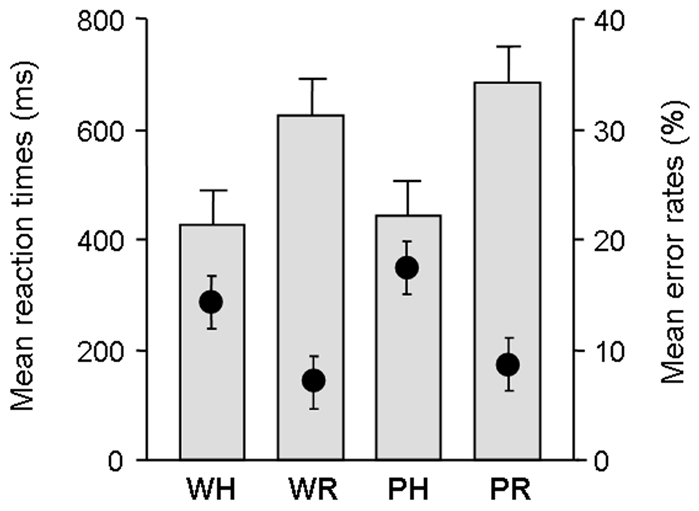
Behavioral results. W refers to Words, P to pseudowords (lexical category under focus), H to Hold and R to Release (sustained attention). Mean reaction times are depicted by histograms and mean error rates are depicted by circles. Error bars indicate standard errors. The reference time for reaction time values is the onset of the stimulus of the pair which has induced the participant's answer, i.e. the onset of the first stimulus of the pair in the ‘release’ condition and the onset of the second stimulus in the ‘hold’ condition.

Behavioural results are depicted in Figure 1. Reaction times in the hold condition were calculated from the onset of the second item of each pair, i.e. 720 ms after the onset of the first item. Error rates were significantly lower in the word than in the pseudoword condition (F[1], [15] = 5.35; p<.05) and in release than hold (F[1], [15] = 11.77; p<.01) with no interaction (F<1; p = .51). Reaction times were significantly faster in the word than in the pseudoword lexical categorization focus condition (F[1], [15] = 12.53; p<.01) and in the hold than in the release attentional condition (F[1], [15] = 64.76; p<.0001), with a significant interaction (F[1], [15] = 6.03; p<.05). Post-hoc *Scheffé* analysis revealed that the advantage of the word condition was only significant in release (word-release versus pseudoword-release, p<.01; word-hold versus pseudoword-hold, p = .61).

**Figure 2:**
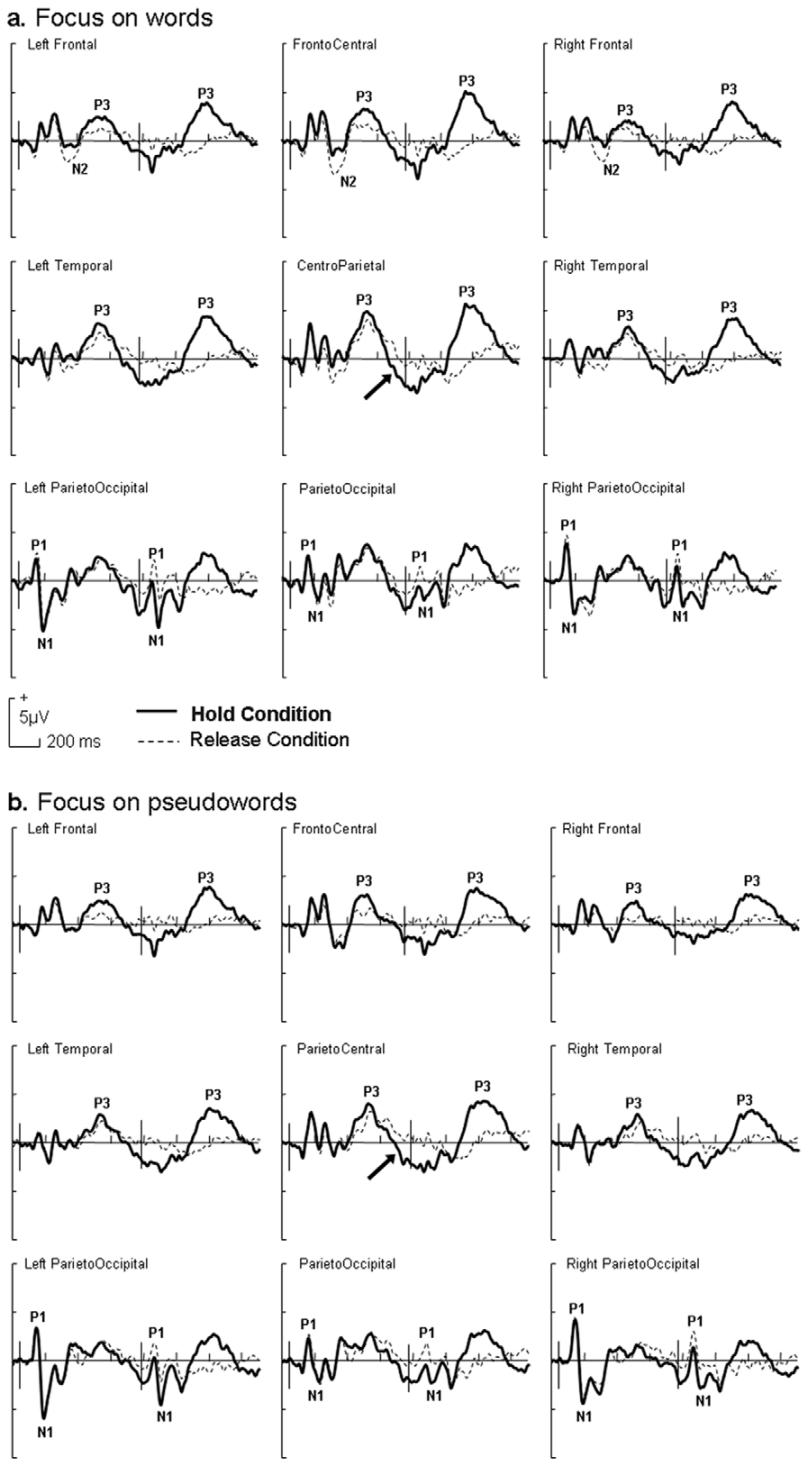
ERP results, general overview. Event-related potential results over nine major scalp regions (Left Frontal, electrodes F3, F5, FC3, FC5, FT7; FrontoCentral, F1, F2, FC1, FCz, FC2; Right Frontal, F4, F6, FC4, FC6, FT8; Left Temporal, C3, C5, CP3, CP5, TP7; ParietoCentral, C1, Cz, C2, CP1, CP2; Right Temporal, C4, C6, CP4, CP6, TP8; Left ParietoOccipital, P3, P5, PO3, PO7, O1; ParietoOccipital, P1, Pz, P2, POz, Oz; Right ParietoOccipital, P4, P6, PO4, PO8, O2). a. Lexical categorization focus on word pairs. b. Lexical categorization focus on pseudoword pairs. ERPs for hold (full line) and release (dotted line) sustained attention conditions. Vertical bars on graphs indicate the onset of the first and second item of a pair.

**Figure 3:**
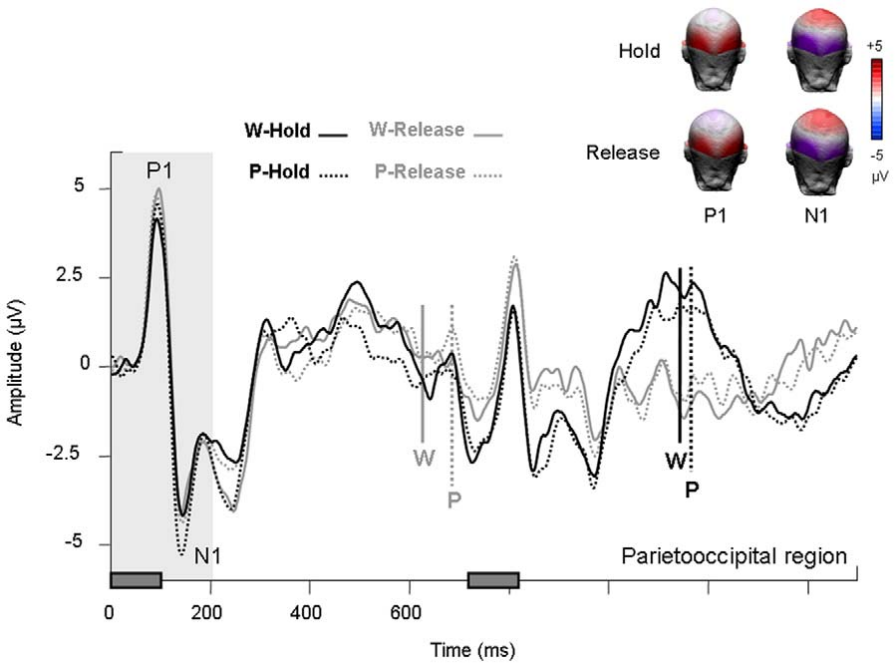
Event-related potential results for the first item of a pair. ERPs measured over parietooccipital region (PO3, PO4, O1, O2, P5, P6, PO7, PO8) for lexical categorization focus and sustained attention conditions (W-hold = words under focus, hold condition; W-release = words under focus, release condition; P-hold = pseudowords under focus, hold condition; P-release = pseudowords under focus, release condition). Rectangles on the time axis indicate the onset and duration of the first and second item of each pair. Vertical bars indicate reaction times.

Three main peaks were observed in the [0–700] ms time window after the first and second stimuli. The P1/N1 complex was observed over bilateral parietooccipital regions and a P3 event was observed over the centroparietal region (see Figure 2). Moreover, we found a slow negativity over centroparietal region between 600 and 800 ms in the hold condition for both word and pseudoword tasks (see arrow over centroparietal region on figure 3) and a N2 event over frontocentral region in the word task – release condition (see frontocentral region on figure 2a).

### ERPs elicited by the first item of a pair

The P1 elicited by the first item peaked at 93±12 ms on average (Figure 3). This peak was significantly more pronounced over the right than the left parietooccipital scalp (F[1], [15] = 9.11; p<.01). Latency and mean amplitude of the P1 were unaffected by lexical categorization focus (latency: F<1; p = .57; mean amplitude: F<1; p = .37) or sustained attention (latency: F<1; p = .43; mean amplitude: F[1], [15] = 3.9; p = .07). The N1 peaked at 139±12 ms on average (Figure 3). This peak was unaffected by lexical categorization focus, sustained attention or recording site.

**Figure 4:**
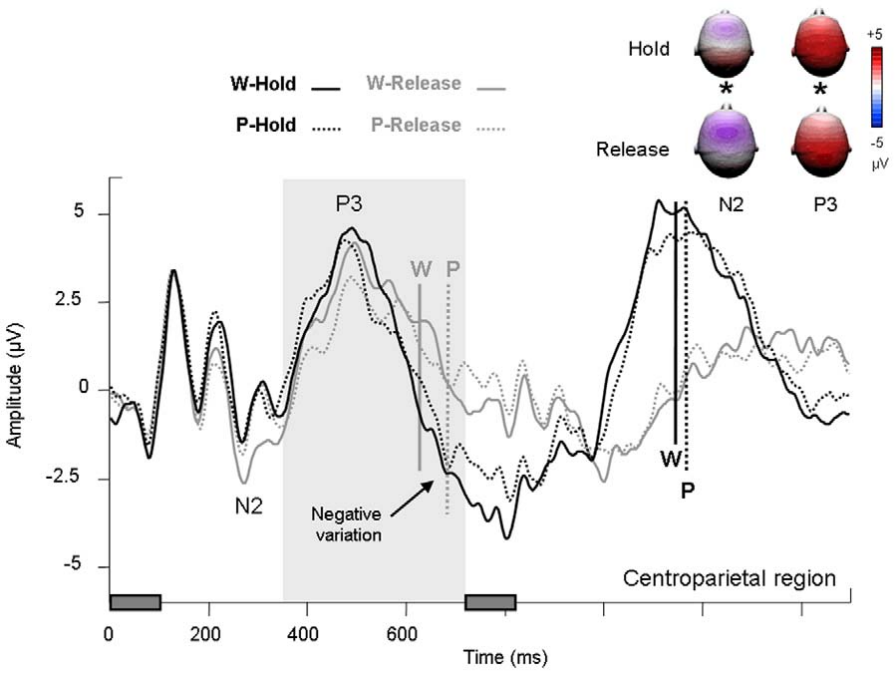
Event-related potential results for the first item of a pair. ERPs measured over centroparietal region (C1, C2, Cz, CP1, CP2, CPz, P1, P2) for lexical categorization focus and sustained attention conditions. Rectangles on the time axis indicate the onset and duration of the first and second item of each pair. Vertical bars indicate reaction times. Topographies labelled with stars are significantly different.

The N2 was maximal over frontocentral region and peaked at 303±31 ms on average (Figure 4). Latency and mean amplitude of the N2 were unaffected by lexical categorization focus (latency: F<1; p = .73; mean amplitude: F[1], [15] = 2.55; p = .13) or sustained attention (latency: F<1; p = .74; mean amplitude: F[1], [15] = 2.16; p = .16). There was no interaction between task and condition in the N2 range (P>.1).

The P3 was maximal over the centroparietal region and peaked at 467±45 ms on average in hold and at 497±59 ms on average in release (Figure 4). The P3 was significantly more pronounced in amplitude in hold as compared to release (F[1], [15] = 10.35; p<.01) and in the word task as compared to the pseudoword task (F[1], [15] = 5.05; p<.05) with no interaction between the two factors (F[1], [15] = 4.22; p = .06). The shape of the P3 wave prevented a meaningful latency analysis in this time-range. Moreover, a negative-going slow wave was observed after the P3 and before the second item was displayed, when participants had to sustain their attention (hold condition; see Figure 4). From 610 ms after the onset of the first item in the word task and from 658 ms in the pseudoword task, the hold ERP shifted toward negative amplitudes, whereas the release ERP remained close to the baseline. An ANOVA was performed on the mean ERP amplitudes between 600 and 800 ms over centroparietal region: The negative variation before the presentation of the second item was significantly larger in amplitude in the word than pseudoword task (F[1], [15] = 4.88; p<.05) and in hold than release (F[1], [15] = 34.47; p<.001) without an interaction (F[1], [15] = 2.09; p = .17).

In sum, the processing of the first item of a pair was unaffected by sustained attention or lexical categorization focus during the first 150 ms (i.e. during the P1/N1 complex). The P3, however, was significantly reduced in amplitude when the participants released their attention, irrespective of lexical categorization focus. The P3 was also increased in the word as compared to the pseudoword task. When participants had to sustain their attention and process the second item of a pair in order to make a decision, the P3 was followed by a negative wave with a centroparietal distribution, which resolved at the onset of the second item.

### ERPs elicited by the second item of a pair

**Figure 5:**
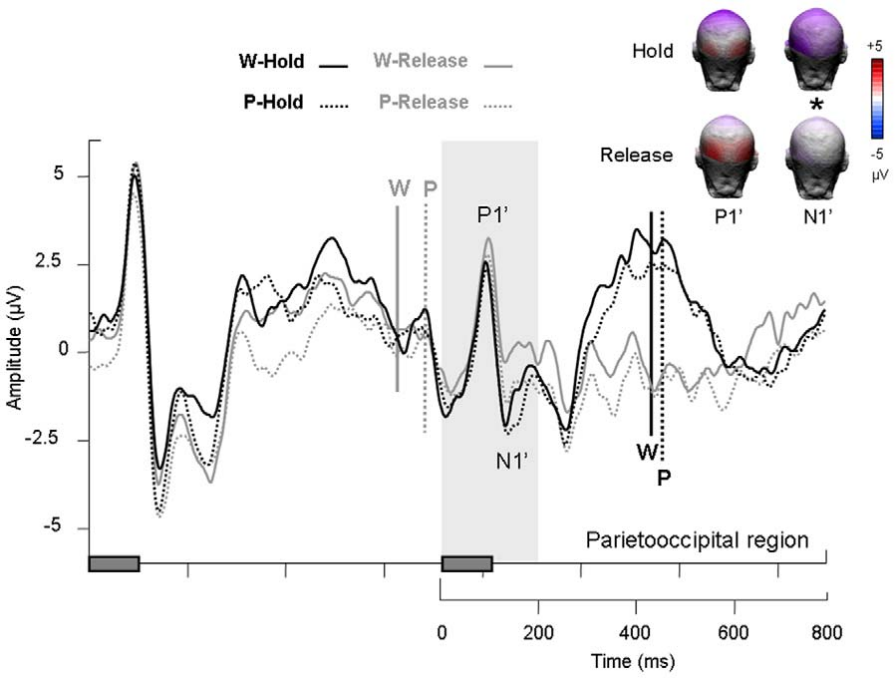
Event-related potential results elicited by the second item of a pair. ERPs measured over parietooccipital region (PO3, PO4, O1, O2, P5, P6, PO7, PO8) for lexical categorization focus and sustained attention conditions. Rectangles on the time axis indicate the onset time and duration of the first and second item of each pair. Vertical bars indicate reaction times. Topographies labelled with stars are significantly different. Time scale and ERP baseline correction were recalculated in reference to the onset of the second item.

**Figure 6:**
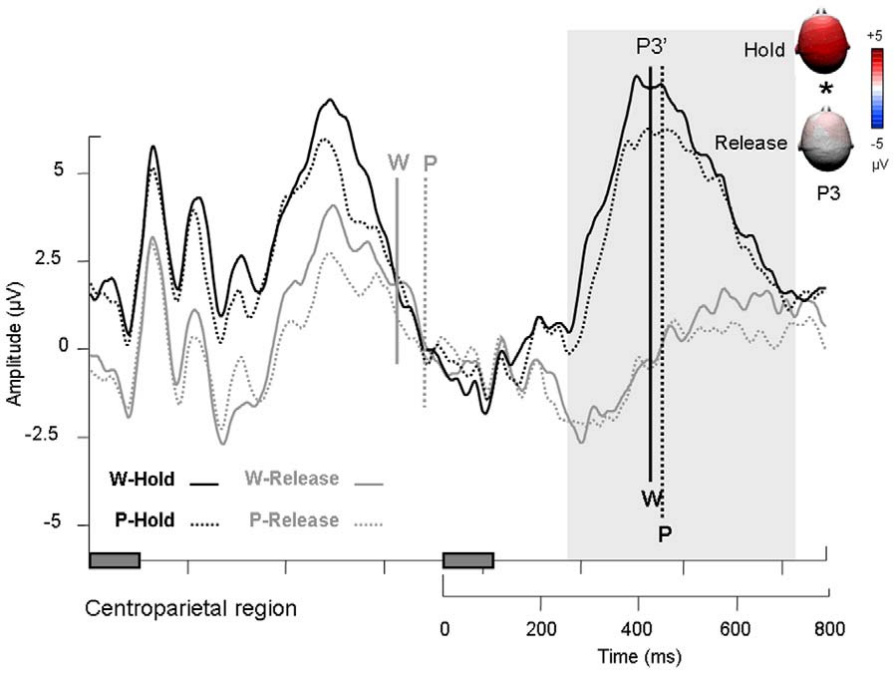
Event-related potential results for the second item of a pair. ERPs measured over centroparietal region (C1, C2, Cz, CP1, CP2, CPz, P1, P2) for lexical categorization focus and sustained attention conditions. Rectangles on the time axis indicate the onset time and duration of the first and second item of each pair. Vertical bars indicate reaction times. Topographies labelled with stars are significantly different. Time scale and ERP baseline correction were recalculated in reference to the onset of the second item.

The first positive component (P1') after the onset of the second item peaked at 85±12 ms on average (Figure 5). The P1' was maximal over parietooccipital region but there was no effect of hemisphere: F[1], [15] = 1.73; p = .21. The P1' was unaffected by lexical categorization focus (F<1; p = .96) or sustained attention (F[1], [15] = 3.74; p = .07) in latency, and there was no interaction (F[1], [15] = 1.44; p = .25). Similarly, P1' mean amplitude was not significantly different between the word and the pseudoword tasks (F<1; p = .75) or between the hold and release conditions (F[1], [15] = 3.29; p = .09), and there was no interaction between the two factors (F<1; p = .55). The N1' peaked at 133±13 ms on average in the hold condition and 139±15 ms in the release condition. It was significantly delayed and reduced in the release as compared to the hold condition (latency: F[1], [15] = 6.11; p<.05; mean amplitude: F[1], [15] = 3.54; p<.05). It was however not affected by lexical categorization focus (latency: F[1], [15] = 3.15; p = .10; mean amplitude: F[1], [15] = 1.85; p = .19) and there was no interaction between lexical categorization focus and sustained attention (latency: F[1], [15] = 1.43; p = .25; mean amplitude: F<1; p = .57). N1' mean amplitude was not different in the two hemispheres (F[1], [15] = 1.61; p = .22). The P3', maximal over centroparietal region, peaked at 447±73 ms on average in the hold condition and was absent in the release condition (Figure 6). P3' mean amplitude was not affected by lexical categorization focus (F[1], [15] = 1.71; p = .21), but it was affected by sustained attention (F[1], [15] = 79.21; p<.0001), without interaction between the two factors (F[1], [15] = 2.14; p = .16). The latency of the P3' was not studied, because there was no identifiable peak in the release condition.

In sum, after the presentation of the second item of a pair, P1', N1' and P3' latencies and mean amplitudes were unaffected by lexical categorization focus (i.e., by the lexical status of the target). When processing of the second item of a pair was not required (release condition), the P1' event tended to be wider and delayed, the N1' event was significantly delayed and smaller and no P3' event was observed. There was no interaction between lexical categorization focus and sustained attention at any stage of processing.

**Figure 7:**
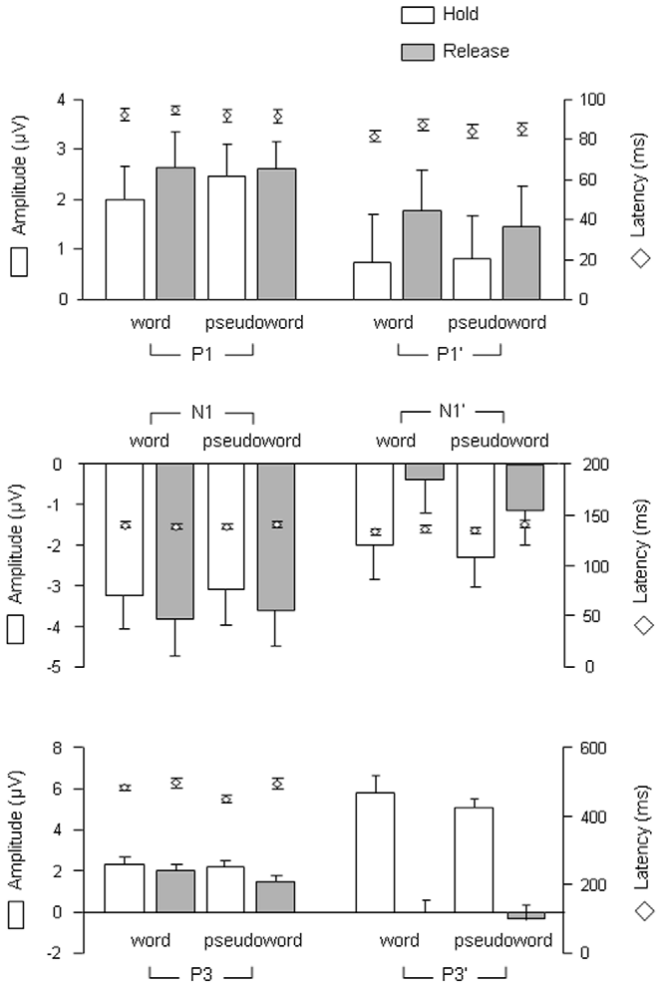
ERP results, summary. Mean amplitudes, mean latencies and standard errors associated with each ERP event in the four experimental conditions (Lexical categorization focus on words or pseudowords; Hold or released sustained attention).

Table 2 and figure 7 summarize the effects of lexical categorization focus and sustained attention on the three ERP events after the presentation of the first and the second items.

**Table 2:** Summary of the effects.

	Sustained attention effect: *Release condition*	Lexical categorization focus effect: *Pseudoword task*
**P1**	n.s.	n.s.
**N1**	n.s.	n.s.
**P3**	reduced	reduced
**P1'**	n.s.	n.s.
**N1'**	delayed and reduced	n.s.
**P3'**	abolished	n.s.

Summary of the effects of sustained attention and lexical categorization focus on the three ERP events after the presentation of the first or the second item of a pair.

## Discussion

### Behavioural results

Participants' reaction times were significantly faster in the hold than release condition whereas error rates showed the opposite pattern. This finding is consistent with a speed-accuracy trade-off effect previously observed in studies using a similar paradigm [1], [19]. Reaction times were shorter in the hold condition probably because participants knew they had to respond to all items in this condition: As long as they had to sustain their attention and process the second item of a pair, participants made a classical lexical decision. In the release condition, however, the situation was more comparable to a ‘go-no go’: After the presentation of the first item, participants had to press a button or withhold their response, depending on the lexical status of the item. This being said, speeded responses in the hold condition appear to have been given at the expense of accuracy.

In the release condition, reaction times and error rates were significantly lower in the word than the pseudoword task, i.e. for rejecting pseudowords when lexical categorization was focused on words. We speculate that in the word task participants might have focussed on lexical access which would have facilitated rejection of non-lexical stimuli (pseudowords). Less efficient processing was observed when participants had to focus lexical categorization processes on pseudowords, which are by definition unfamiliar: no preparation was possible when participants had to focus on these stimuli which do not match any entry in memory.

### P1/N1 complex and lexical categorization focus

The P1/N1 complex appeared to be unaffected by lexical categorization focus in both the case of the first and the second item (cf. Figure 7). Therefore, early sensory analysis (thought to be reflected by P1; see [24]) and visual discrimination processes (thought to be reflected by N1) were not modulated differentially when participants focussed on words or pseudowords in the lexical categorization processing, despite the fact that both error rates and reaction times were lower for words than for pseudowords. Interestingly, facilitation in letter-string processing has been observed as early as the N1 range when words were compared with unpronounceable non-words (i.e., consonant strings; [25]). Therefore, within the N1 range, lexical categorization focus may affect word versus consonant string discrimination but not word versus pronounceable pseudoword discrimination. Therefore the process indexed by the N1 seems insensitive to stimulus lexicality when written sequences are word-like.

### P1/N1 complex and sustained attention

Interestingly, the P1/N1 complex was observed after the presentation of the second item of a pair in both the hold and release conditions. Thus, P1 event (indexing early sensory analysis of visual information; [26]) and N1 event (reflecting visual discrimination processes; [25]) appear to relate to automatic aspects of perceptual processing since they are little affected by sustained attention. Nevertheless, the P1 tended to be wider and delayed in the release condition after the presentation of the second item. Also, the N1 elicited by the second stimulus was significantly reduced and delayed in the release condition (cf. Figure 7). Thus, the presence of a P1/N1 complex probably reflects automatic processes modulated by sustained attention (at least in the case of the lexical decision task used here; cf. [13], [27]).

### P3 and categorical and attentional effects

The P3 was significantly reduced in the pseudoword task as compared to the word task in the case of the first item, but not in the case of the second. Moreover, the P3 was significantly reduced in the release as compared to the hold condition after the first item, and was abolished in the release condition after the second item. Taking the view that the P3 reflects information updating in working memory [28], [29], it is logical that it should be affected by both lexical categorization focus and sustained attention. When participants have to release their attention after integrating the first item, working memory updating is likely to operate differently as in the hold condition. In fact, since the P3 was cancelled in the case of the second item in the release condition, it can be assumed that working memory update did not take place at all in this condition. This assumption is congruent with Schupp et al.'s comment on the fact that sustained attention is reflected by sustained positive slow waves [16]: in fact, the authors propose that “the call for processing resources triggered after initial stimulus categorisation assures that attended stimuli have priority access to a capacity-limited stage required for working memory consolidation and conscious recognition”. Note that the P3 event was unlikely to be accounted for by motor action or response decision because its mean amplitude was not correlated to reaction times (R^2^_(WR)_ = .03; R^2^_(PR)_ = .08; R^2^_(WH)_ = .63; R^2^_(PH)_ = .30).

In the Release condition, the P3 amplitude was marginally smaller in the Pseudoword than in the Word task. This result is consistent with the interaction found in RTs between task and condition (RTs were shorter in the Word than in the Pseudoword task in the release condition). In other words, focusing on words facilitates rejection of non lexical stimuli after working memory update. In contrast, focusing on pseudowords would load working memory to a lower extent and lead to longer RTs.

### Attentional blink

When the amount of time between two targets requiring report (T1 and T2) is short enough, response to T2 –whatever the task– is less accurate or slower, as revealed by the typical results of the psychological refractory period paradigm [30] and the attentional blink (AB) paradigm [31], [32]. The AB phenomenon has been described as a transitory impairment of attention in the temporal domain. Using the AB paradigm, a variety of ERP components have been studied to determine the first stage at which processing is altered or suppressed [32], [33]. No changes in amplitude or latency were found in the P1 or N1 ranges. However, the P3 wave was completely suppressed during the period that corresponds to the AB. This observation is consistent with the idea that the AB occurs after the initial perceptual stage is over and that it reflects a failure to store T2 in working memory while T1 is being analysed [34], [35]. Even though the present experiment did not involve rapid serial visual presentation of stimuli, some parallels may be drawn between the Hold/Release and the AB paradigm: ERP results observed in AB paradigms are similar to the results of the present study in the release condition, although the effect was prompted by participants' strategy during the task rather than stimulus-driven: When processing of our “T2” (the second stimulus of a pair) was not necessary, P1 and N1 components were affected but still present whereas the P3 was suppressed.

### Contingent negative variation

In addition to the classical P3 ERP component, we observed a negative variation in the interval between items 1 and 2 (Figure 5). This variation was significantly larger in hold than release and is reminiscent of the processing negativity [36], [37] or contingent negative variation (CNV; see [38], [39]), previously considered to reflect working memory engagement in linguistic tasks [1], [10], [17], [40] but also attentional effects [41], [42], [43], [44]. Interestingly, in the present experiment, the time interval between items 1 and 2 was not different from intervals traditionally used in Go/No-go paradigms: After responding to the first item of a pair, participants had to release their attention. In this condition (Release), ERP waves remained close to the baseline, reflecting the withdrawal of further preparation. Similar ERP waves have been observed in the No-go condition of Go/No-go studies [45], [46], [47], [48]. When participants had to wait for the presentation of the second item of the pair, they had to sustain their attention between the presentation of item 1 and 2. In this condition (Hold), ERP waves displayed a typical pattern of slowly increasing negativity presumably reflecting the build-up of attentional resources necessary for adequate processing of the following item [49]. Similar ERP waves have been reported in the Go condition and Go/No-go studies [45], [46], [47], [48]. Thus, in the present study as in Go/No-go experiments, the negative variation in the interval between items 1 and 2 can be interpreted as a correlate of sustained attention load.

### Conclusion

In the present study, we explored the effects of sustained attention and lexical categorization focus during a visual lexical decision task. Early sensory analysis and visual discrimination of words and pseudowords, reflected by the P1/N1 ERP complex, were unaffected by the lexical categorization focus. Moreover, this processing stage appeared to be mostly ‘automatic’ since disengagement of sustained attention modulated the P1/N1 complex without completely ‘blocking’ it. By contrast, working memory updating was highly dependent upon the allocation of sustained attention, i.e., it was ‘blocked’ when sustained attention was released. Indeed, the P3 wave considered as an index of working memory updating was enhanced and followed by a slow negativity when attention was sustained. Further studies will specify the effects of inter-stimulus interval and working memory load on these processes. The Hold/Release paradigm seems to be a promising paradigm to explore the influence of sustained attention in the processing of stimuli presented in a serial fashion.

## Materials and Methods

### Ethics Statement

All participants gave written informed consent to participate in the experiment that was approved by the ethics committee of Midi-Pyrenees, France.

### Participants

Sixteen French native speakers (8 females and 8 males; mean age 21.7±2.1 years, all right-handed) gave informed consent to participate in the experiment. All participants had normal or corrected-to-normal vision.

### Stimuli

Stimuli were 80 French nouns (e.g., CREME; ‘CREAM’) selected from the Brulex database [50] and 80 pseudowords (e.g., CTEME; pronounceable nonwords made by switching or replacing 1 or 2 letters within the first syllable of each noun, so as to obtain an aberrant digraph under the optimal viewing position; see [51], [52]). Mean string length was 5.65±0.48 letters (range 5–6 letters) and mean log converted lexical frequency of nouns was 3.41 (range 3.11–3.70). Four groups of stimulus pairs were pseudo-randomly generated avoiding semantic or phonological links (word-word, word-pseudoword, pseudoword-word, pseudoword-pseudoword).

### Task and procedure

**Figure 8:**
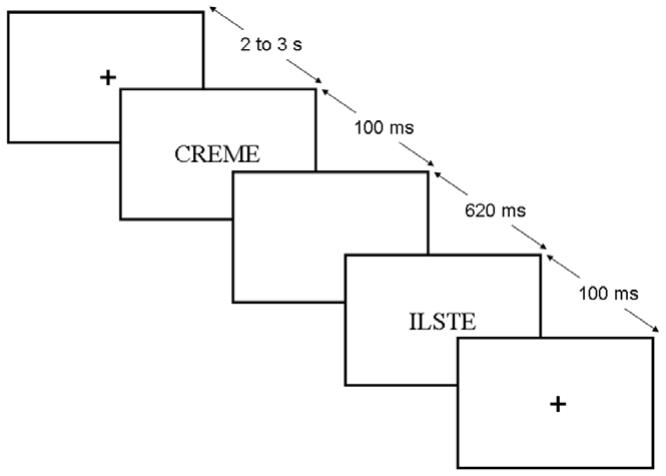
Experimental design: Example of one trial.

The experiment was divided in two parts: (a) In the word task participants had to press a designated button for stimulus pairs comprising two real words (e.g., *RUINE – LISTE*; ‘RUIN – LIST’) and another button for every other combination (word – pseudoword; pseudoword – word; pseudoword – pseudoword; e.g., *CREME* – ILSTE; ILSTE – *CREME*; CTEME – ILSTE). (b) In the pseudoword task, targets were stimulus pairs comprising two pseudowords (e.g., CTEME – ILSTE) as opposed to other combinations involving words (pseudoword – word; word – pseudoword; word – word). Each participant performed both tasks and task order was counterbalanced between participants. In each trial, two different situations could arise: (1) when the first item of a pair was congruent with the target category, participants had to sustain their attention and hold the lexical category of the first item of the pair in working memory until they could reach a decision about its congruency (hold condition). (2) When the first item of a pair was incongruent with the target category, participants could respond “no” immediately without waiting for the second item (release condition). Experimental conditions are summarised in Table 2. Four blocks of 40 stimulus pairs (10 pairs of each category) were presented in each of the two tasks. The stimuli were displayed at fixation for 100 ms separated by a 620 ms pause (blank screen). Inter-pair interval was variable (2000–3000 ms) and allowed for participant's response (see Figure 8). Each individual stimulus was used once in first and second position in the word task and once in first and second position in the pseudoword task. Response sides were counterbalanced across blocks and participants.

### ERP acquisition and processing

Electrophysiological data were recorded from 64 Ag/AgCl electrodes (placed according to the extended International 10–20-system) at a sampling rate of 500 Hz, using SynAmps™ amplifiers (Neuroscan™, El Paso, TX, USA). Signals were filtered on-line between 0.1 and 100 Hz. Impedances were kept below 20 kOhms. Continuous recordings were digitally band-pass filtered off-line in the interval [1]-[40] Hz. Eye blink artifacts were mathematically corrected based on a model artifact computed from a minimum of 50 individual artifacts in each participant using the procedure implemented in Scan 4.3 (Neuroscan™, Inc., El Paso, TX, USA) and remaining artifacts were manually dismissed. Epochs ranged from -100 to 1500 ms after the onset of the first stimulus of each pair. After baseline correction relative to pre-stimulus activity ([−100; 0] ms for the first item analysis and [620; 720] ms for the second item analysis) and rejection of errors, there was a minimum of 40 epochs per condition in each participant. Individual difference waveforms and grand-average waveforms were then derived from individual ERPs. Artifacts on the ERP signal due to responses during the second stimulus display were not corrected because the P1' component was rarely recorded simultaneously with motor response.

### Statistical analysis

Behavioural results (error rates and reaction times) were analysed statistically by means of a repeated measure analysis of variance (ANOVA). The two main factors were sustained attention (hold versus release) and lexical categorization focus (searching for word versus pseudoword pairs). To correct for sphericity violation, we applied a Greenhouse-Geisser correction [53]. Reaction times were measured starting from the display of the item of the pair which induced the participant's response, i.e. time 0 corresponded to the display of the first item of the pair in the release condition and that of the second item of the pair in the hold condition.

ERP peak search was confined to specific intervals on the basis of the main components identified on the Mean Global Field Power of all 64 electrodes [54]. Peaks elicited by the first item of a pair were detected in the following intervals: 60 to 114 ms for the P1, 114 to 170 ms for the N1, 250 to 350 ms for the N2 and 370 to 590 ms for the P3. For the second item of a pair, search intervals were 60 to 110 ms for the P1, 110 to 156 ms for the N1 and 310 to 560 ms for the P3 (in reference to the onset of the second stimulus). P1 and N1 were studied at 16 parietooccipital electrodes where peak amplitude was maximal, P3 was studied at 12 centroparietal electrodes and N2 was studied at 14 frontocentral electrodes. Mean peak amplitudes and latencies were analysed for each component using a repeated measures ANOVA. ANOVA factors were sustained attention (hold vs. release), lexical categorization focus (word vs. pseudoword) and hemisphere (left vs. right).
